# A qualitative investigation of the role of paediatric rehabilitation professionals in rural South Africa: Rehabilitation professionals’ perspectives

**DOI:** 10.4102/sajp.v72i1.290

**Published:** 2016-02-26

**Authors:** Desmond Mathye, Carina Eksteen

**Affiliations:** 1 Department of Physiotherapy, University of Pretoria, South Africa

## Abstract

**Purpose:**

To investigate the role that rehabilitation professionals play in the rehabilitation of children with disabilities in the rural and under-resourced community of Giyani in South Africa.

**Method:**

A qualitative, exploratory and descriptive approach was used. Semi-structured face-to-face interviews were used to collect data from a convenient sample of eight rehabilitation professionals. Data were transcribed verbatim by two trained students and verified by the main researcher. An inductive approach to qualitative data analysis was used. *In vivo* and open coding were used to generate codes.

**Results:**

Analysis of data resulted in 21 codes, 9 subcategories, 5 categories and 1 theme. The role of rehabilitation professionals was described in terms of the five categories which are to *examine* newborn babies and children at risk, *support* caregivers of children with disabilities, impart *skills training* for caregivers of children with disabilities, *rehabilitate* children with disabilities and conduct *follow-ups* in communities where the children with disabilities reside.

**Conclusion:**

The role that rehabilitation professionals play in the rural and under-resourced community of Giyani in South Africa is similar to the role played in high-income countries. The role that rehabilitation professionals play is not only focused on the child but also on the family.

## Introduction

Physiotherapists and occupational therapists play various roles in the rehabilitation of children with disabilities. They not only assist children with disabilities in rehabilitation but also help families and caregivers of children with disabilities with necessary skills to enable the disabled children to participate in society (Chiarello *et al*. 2011). Of all the rehabilitation professionals rendering rehabilitation services to children with disabilities in South America (Brazil), physiotherapists and occupational therapists were found to perform the most comprehensive assessments of children and treatment, making them the most relevant professionals to conduct rehabilitation of children with disabilities (Andrade *et al*. 2012).

Physiotherapists, occupational therapists and speech therapists are traditionally referred to as ‘rehabilitation professionals’ (Chiarello *et al*. 2011; Levin 2006). The rehabilitation of children with disabilities requires the concerted effort of the multidisciplinary team of a physiotherapist, occupational therapist, speech therapist, nurses, psychologist and others (Jeglinsky, Autti-Rämö & Brogren Carlberg 2012). The different health professionals play various roles in the rehabilitation of children with disabilities, and at times, they became part of ‘rehabilitation professionals’ even though they do not meet the definition of rehabilitation professionals as suggested by some authors (Chiarello *et al*. 2011; Levin 2006). For example, some authors reported that nurses also have a role to play in the rehabilitation of children with disabilities (Olli, Vehkakoski & Salanterä 2014).

Rehabilitation professionals are best known for the ‘hands-on’ role that they play in the rehabilitation of children with disabilities such as soft tissue mobilisation, joint mobilisation, positioning and others (Levin 2006). Nevertheless, by only focusing on the ‘hands-on’ role, rehabilitation professionals do not sufficiently address the broader challenges facing children with disabilities and their families such as their educational needs and social security (Egilson 2011; Levin 2006; Saloojee *et al*. 2007). One study has suggested that paediatric rehabilitation professionals play a significant role in the lives of children with disabilities when working in partnership with the family or caregivers of such children (An & Palisano 2014). It was also suggested that rehabilitation professionals, in exercising their responsibilities towards children with disabilities, should focus on family-identified needs, shared responsibility and family empowerment (An & Palisano 2014). However, some authors suggest that the rehabilitation of children with disability should shift from a problem-oriented, therapist-directed approach to a possibilities-oriented approach or coaching where client empowerment takes precedence (Baldwin *et al*. 2013). Nonetheless, it has been acknowledged that coaching requires families to self-reflect and to do critical thinking, which might not fit in some cultural contexts around the world (Baldwin *et al*. 2013).

The role that rehabilitation professionals play in the rehabilitation of children with disabilities in high-income countries has been fully documented in the literature (Baker *et al*. 2012; Effgen, Chiarello & Milbourne 2007; Egilson 2011; Wiart *et al*. 2010). However, literature that specifically dealt with the role of paediatric rehabilitation professionals in low- to middle-income countries like South Africa could not be found.

The purpose of this study is to investigate the role that rehabilitation professionals play in the rehabilitation of children with disabilities in the rural and under-resourced community of Giyani in the Limpopo Province of South Africa. Our objective is to identify and describe the role of paediatric rehabilitation professionals from the perspectives of the rehabilitation professionals.

## Research design

### Methods

#### Study setting

This article is part of a larger study that was conducted in the Greater Giyani Municipality in the Limpopo Province of South Africa where the primary aim was to develop a model for the rehabilitation of children with disabilities. The Greater Giyani consists of 10 traditional authorities, 91 villages and a township (Giyani). Giyani used to be the capital of the former Gazankulu Government. It is now the administrative and commercial centre of both the Mopani District Municipality and the Greater Giyani Municipality (Local Government Handbook of South Africa [LGHSA] 2013), with a population of about 244 217 people (Statistics South Africa [STATSSA] 2012).

## Study design

A qualitative, exploratory and descriptive approach was used to obtain the participants’ perspectives on the role of rehabilitation professionals. A qualitative research is used to collect data in a face-to-face situation by interacting with participants in their own setting or context, with the aim of understanding the participants’ meaning of events from their own perspectives (McMillan & Schumacher 2006). Considering that literature on the role of rehabilitation professionals in a rural South African context could not be found, an exploratory and descriptive approach was used to examine the ‘little-understood’ phenomena in order to develop a detailed concept (Babbie & Mouton 2001; McMillan & Schumacher 2006). Data were collected from a convenient sample of rehabilitation professionals working at Nkhensani Hospital in Giyani.

### Participant selection

Rehabilitation professionals were directly and indirectly invited to participate in the study. The researcher circulated the study’s information leaflets directly and indirectly (through others) to all rehabilitation professionals in the physiotherapy, occupational therapy, speech therapy and the nursing departments. The rehabilitation professionals who received the information leaflets in person were given the opportunity to ask questions. Rehabilitation professionals who received the information leaflet from their colleagues had an option of calling or texting the researcher if they needed clarity.

### Sample size and inclusion criteria

In total, staff members from physiotherapy (*n* = 6), occupational therapy (*n* = 5), speech therapy (*n* = 1) and nursing (*n* = 1) were invited to participate in the study. To be included in the study, participants’ had to be registered as a physiotherapist, occupational therapist, speech therapist or a therapy assistant with the Health Professions Council of South Africa or registered as a nurse with the South African Nursing Council. In addition to being registered with the statutory bodies, participants must have worked with disabled children at Nkhensani Hospital.

The participants who signed an informed consent form were from the physiotherapy (*n* = 2), occupational therapy (*n* = 5) and nursing (*n* = 1) departments. The demographic characteristics of all participants are as presented in Table 1.

**TABLE 1:** Demographic characteristics of participants.

Code	Age	Gender	Occupation	Qualification	Experience
Prof 1	46	Male	Occupational therapy assistant	Diploma in OTA (Community)	10 years
Prof 2	26	Female	Occupational therapist	B.Occ.Ther.	3 years
Prof 3	47	Male	Physiotherapy therapy assistant	Registered with Health Professions Council of South Africa (no formal qualification)	25 years
Prof 4	23	Female	Occupational therapist (community service)	B.Occ.Ther.	5 months
Prof 5	30	Male	Physiotherapist (community service)	BSc (Physio.)	5 months
Prof 6	43	Female	Occupational therapy assistant	Diploma in OTA (Community)	10 years
Prof 7	26	Female	Occupational therapy assistant	Diploma in OTA (Community)	4 years
Prof 8	56	Female	Professional nurse	B.Cur., M.Cur.	35 years

### Data collection procedure

Semi-structured interviews were used to collect data from the eight rehabilitation professionals. All interviews were conducted at Nkhensani Hospital at a time that participants preferred. On the day of the interview, the main researcher ensured that participants were free and comfortable by cracking some jokes as he began setting up the recording devices. The researcher started the interview by greeting and introducing himself. Participants were asked what their role was in the rehabilitation of children with disabilities in Giyani. Two digital audio recorders were used simultaneously to record all interviews. The two audio recorders were simultaneously used just in case one failed. Interviews were carried out in English or vernacular language (Xitsonga), depending on the participants’ preference. Nonetheless, there were times when responses were in English, Xitsonga and Tshivenda. At the end of the interview, the researcher thanked the participant and made an appointment for a follow-up interview based on the participant’s preference. Follow-up interviews were conducted with seven of the eight participants.

### Ethical consideration

The permission to conduct this study was approved by the Ethics Committee of the Faculty of Health Sciences of the University of Pretoria (Protocol 109/2009). In addition, the study was supported by the Limpopo Provincial Department of Health and Social Development. All participants in this study signed an informed consent form.

### Strategies to ensure trustworthiness

Trustworthiness refers to the activities that were implemented to ensure that qualitative data were gathered and analysed rigorously so as to ensure that the outcome of the research is correct (Speziale & Carpenter 2007). Credibility, dependability, confirmability and transferability are the four criteria used to demonstrate trustworthiness of qualitative research (Lincoln & Guba 1985). For the purpose of this article, measures used to ensure the credibility and transferability of the study are shown.

### Measures used to ensure credibility

The background and experience of the researcher were used to ensure the credibility of the study (Shenton 2004). The researcher has been living in the Giyani area for over 10 years, understood the local language and culture and has been practicing as a physiotherapist for over 10 years before embarking on the current study. In addition, the researcher had a prolonged engagement (Lincoln & Guba 1985) at Nkhensani Hospital where he was able to be acquainted with some of the therapists. Lastly, a literature review was conducted after data analysis so as to assess the degree to which the research results are congruent with the results from the latest literature.

### Measures used to ensure transferability

According to Babbie and Mouton (2001), transferability is the extent to which the findings of the study can be applied in other contexts or with other participants. Even though the aim of the current study was not to generalise the findings, it is expected that the findings will have relevance to participants in a similar situation (Speziale & Carpenter 2007). A detailed presentation of the methodology of the study will enable the reader to find meaning of the present study in the studies of a similar context.

### Analysis of data

Data generated from the interviews were transcribed verbatim by two trained senior university students who were trained by the main researcher to be data transcribers. Verbatim transcripts were verified and corrected by the researcher who later translated them into English in an event that Xitsonga and Tshivenda were used during the interviews. All transcripts were uploaded onto a qualitative data analysis programme (*Atlas.ti v6*).

The *Atlas.ti* programme was only used to store, organise and reconfigure data. It is the researcher who coded data, not *Atlas.ti* programme (Saldana 2013). An inductive approach to qualitative data analysis was used (Thomas 2006). The inductive approach is not associated with any individual qualitative approach but is generic in nature and may be applied to different types of research approaches (Ezzy 2002; Silverman 2000).

*In vivo* and open coding were used to generate codes. *In vivo* coding is the creation of a code from the selected text, such as the uploaded transcripts (Muhr 2004). *In vivo* coding is defined as a code that is taken directly from the participant using his or her words (Saldana 2013). On the other hand, open coding is when a qualitative researcher creates, names and lists a code (Muhr 2004).

## Results

The theme that emerged from the analysis of data is ‘the role of rehabilitation professionals in the rehabilitation of children with disabilities’. The theme comprises five categories, nine subcategories and 21 codes. Each of the five categories was derived from subcategories, which were in turn derived from the codes assigned from the participants’ quotations.

The role that rehabilitation professionals play in the rehabilitation of children with disabilities was described in terms of the five categories: *examine*, *support*, *skills training*, *rehabilitate* and *follow-up*.

### Definition of categories

The terminologies used to describe the categories in this study can also be used in other or different contexts. For the purpose of this article, these terminologies have been defined in order to give meaning that is specific for this research context.

According to the Oxford dictionary, to *examine* is to look at somebody closely, to see if there is anything wrong or to find the cause of a problem (Hornby 2000). For the purpose of this article, *examine* refers to the screening of a child, asking the caregiver about the child’s condition and physically touching the child to establish what is wrong.

*Support* has multiple definitions in the literature, but the Oxford definition which is similar in definition to the study context defines it as to help or encourage somebody such as the caregiver by saying or showing (e.g. through counselling) that you agree with them or understand their situation or condition (Hornby 2000:1359).

*Skills’ training* is the teaching of specific verbal and nonverbal behaviours and the practising of these behaviours by the patient (Mosby’s Medical Dictionary 2009). *Skills training* in the current study meant teaching and/or showing caregivers by rehabilitation professionals the necessary skills to ‘treat’, handle or manage a disabled child (Mosby’s Medical Dictionary 2009).

The World Report on Disability defines rehabilitation as a set of measures that assist individuals who experience or are likely to experience disability to achieve and maintain optimal functioning in interaction with their environment (World Health Organization [WHO] 2011). For the purpose of this study, *rehabilitate* refers to a series of steps taken (e.g. hands-on therapy, issuing of assistive devices and home programme), with the aim at helping children who acquired disabilities before, during and after birth to enable them to regain maximal functioning.

The context under which *follow-up* was used in this study is similar to the Oxford dictionary definition. According to the Oxford definition, *follow-up* is an action that continues something that has already started (Hornby 2000:529), such as the rehabilitation of children with disabilities as was the case in this study.

### Description of results

#### ‘Examine’ – children with disabilities

Participants (all therapy assistants) have suggested that the role of rehabilitation professionals and, in particular, a nurse (acting as a rehabilitation professional as was the case in this study) is to assess and screen babies for any trace of disabilities. Three rehabilitation professionals have concurred that a nurse (acting as rehabilitation professional) has a role in the examination of children with disabilities, as shown in Table 2.

**TABLE 2:** Role of rehabilitation professionals: Examine.

Category	Subcategory	Code	Quotation
Examine	Screen and assess	Screen newborn babies Test babies Assess	‘Sister S screen newborn babies from the maternity ward and she identify defects in the children’. (Prof. 3) ‘What we know is that she screens and tests babies for genetic problems immediately after birth at the maternity ward’. (Prof. 1) ‘She also checks where the problem comes from, whether it has been inherited or not’. (Prof. 6)

#### ‘Support’ – caregivers of children with disabilities

Four participants have suggested that the role of rehabilitation professionals is to *counsel* and *support* mothers or caregivers of children with disabilities as shown in Table 3.

**TABLE 3:** Role of rehabilitation professionals: Support.

Category	Subcategory	Code	Quotation
Support	Counsel	Counsel	‘We tell them to accept their condition that it is not a sin to get a disabled child, and we counsel them’. (Prof. 1) ‘We also counsel’. (Prof. 6) ‘We assist them with counselling … we request those mothers to come back to us for continuous counselling … and to meet other mothers where they share their problems and resources’. (Prof. 8)
	Support	Encourage Provide support	‘We also encourage them not to stop coming because change takes time’. (Prof. 1) ‘We encourage them to rehabilitate their kids because as mothers; they are the ones who spend most of the time with the kids than us … giving the mother and child emotional support’. (Prof. 2) ’We encourage, and support [caregivers]’. (Prof. 6)

#### ‘Skills training’ – for caregivers of children with disability

Seven participants have indicated specific roles that they perform as they impart skills training for the caregivers, such as to teach, educate, explain, train and guide as indicated in Table 4.

**TABLE 4:** Role of rehabilitation professionals: Skills training.

Category	Subcategory	Code	Quotation
Skills training	Education	Teach Educate Explain Train Guide	‘We also teach the mothers how to handle the kids at home as well as exercising them’. (Prof. 3) ‘We teach the caregivers’. (Prof. 4) ‘Our other role is to educate the mothers about their children’s conditions’. (Prof. 5) ‘We explain to them what their children or grandchildren’s conditions are’. (Prof. 1) ‘We try to explain to them and encourage them to rehabilitate their kids’. (Prof. 2) ‘And train the caregivers on how to care for their children’. (Prof. 6) ‘We are just here to guide them with the expertise that we have’. (Prof. 7)

#### ‘Rehabilitate’ – children with disabilities

The *rehabilitate* category consists of four subcategories: -*hands-on roles, issue assistive device, refer* and *home programme*. Participants have reported on some roles that rehabilitation professionals play in the rehabilitation of children with disabilities. These particular roles include the *hands-on role*, where rehabilitation professionals *use different techniques to treat*, *facilitate, stretch and mobilise* children with disabilities. Some of the participants have reported that rehabilitation professionals also *issue assistive devices* and have the responsibility to *refer* children with disabilities to other healthcare professionals when necessary. Other participants have suggested that rehabilitation professionals issue *home programme* to the families or caregivers of children with disabilities, as summarised in Table 5.

**TABLE 5:** Role of rehabilitation professionals: Rehabilitate.

Category	Subcategory	Code	Quotation
Rehabilitate	Hands-on role	Mobilise child Facilitate Stretch Treat Use different techniques	‘We use different kinds of techniques to treat them for their development’. (Prof. 4) ‘We are offering rehabilitation services where we mobilise children and facilitate.… Some children come with contractures, and we stretch those contractures using back slabs’. (Prof. 3)
	Issue assistive device	Assistive device	‘Issuing assistive devices’. (Prof. 2)
	Refer	Refer	‘We refer kids to them [other professionals]’. (Prof. 3) ‘If we assess the child and find that there is a hearing or eye problem, we refer to the speech therapist or optometrist’. (Prof. 5) ‘We do refer clients to those other disciplines … when we identify the social problem; we refer them to social workers or straight to the doctors who will assist them with a disability grant’. (Prof. 1) ‘Referring them to relevant health professionals, for example, dieticians and dental practitioners’. (Prof. 2) ‘We refer to other health professionals based on the needs that we have identified’. (Prof. 8)
	Home programme	Home programme	‘We then show them home programmes’. (Prof’ 3) ‘[We] ask the caregivers if they are performing the home programme that we gave to them’. (Prof. 5) ’We teach the mothers of these children to do home programmes such as positioning’. (Prof. 1) ‘We teach the caregivers as well so that they can do the home programme when they are at home’. (Prof. 4)

#### ‘Follow-ups’ – on children with disabilities

Two participants have reported that rehabilitation professionals do *follow-ups* and conduct *home visits* in the communities where children with disabilities and their caregivers reside, as depicted in Table 6.

**TABLE 6:** Role of rehabilitation professionals: Follow-up.

Category	Subcategory	Code	Quotation
Follow-up	Conduct home visits	Home visits Home follow-up	‘We also do home visits where we do follow-ups on what we have showed them in the hospital’. (Prof. 3) ‘After discharging them, we do follow-ups at home or refer them to CRWs, who will do home visits. We also do home visits from the hospital’. (Prof. 1)

## Discussions

The focus of this article was to highlight the rehabilitation professional’s perspective about their role in the rehabilitation of children with disabilities in the rural community of Giyani in Limpopo Province, South Africa. The role of rehabilitation professionals in the rehabilitation of children with disabilities emerged from the analysis of data as a major theme, consisting of five categories: (1) *examine*, (2) *support*, (3) *skills training*, (4) *rehabilitate* and (5) *follow-up*. The examination of children with disabilities in the context of this study includes screening and assessment of babies by a nurse. However, the focus of the examination is mainly directed at identifying biological characteristics of the disabled child and not on the overall health-related quality of life. Even though it is understood that children with disabilities would not be able to state their general health-related quality of life, it is possible to obtain the information by proxies from caregivers and other healthcare professionals (Morrow *et al*. 2012).The International Classification of Functioning, Disability and Health model that guides healthcare professionals on how to examine, assess and plan the management of children with disabilities cautions against focusing only on biological characteristics of the child. Instead, the International Classification of Functioning, Disability and Health guides health professionals towards a comprehensive examination of health, including the biological, individual and social perspective of the child (Stucki 2005; WHO 2001). Nonetheless, the findings of this study on the *examine* category is in line with another study, which suggests that screening is one of the most important tool used in identifying children with disabilities and is the first step in providing intervention (Yousafzai, Lynch & Gladstone 2014). Another study has also reported that the role of rehabilitation professionals include screening and assessment of children with disabilities (Bunning *et al*. 2014).

It has been widely reported in the literature that caregivers of children with disabilities are subjected to high levels of stress (Dabrowska & Pisula 2010; Hayes & Watson 2013; Parkes *et al*. 2011), and they require some form of support and counselling in order to reduce it (Raina *et al*. 2005). As such, the category of *support*, as part of the role of rehabilitation professionals as expressed in this study is in line with what was found by other researchers.

The need for rehabilitation professionals in providing *skills training* for caregivers of children with disabilities is not unique to this study as it was also reported by Einfeld *et al*. (2012). Considering that the rehabilitation of children with disabilities is not a once-off event, but a lengthy process that happens both in the hospital and at home. It is, therefore, essential to equip caregivers with the necessary skills to enable them to continue with the rehabilitation at home. Skills training is cost-effective in the long run whilst taking into consideration the shortage of health professionals in low- to middle-income countries (Einfeld *et al*. 2012) and, in particular, rural areas like Giyani in South Africa.

Participants have also indicated that the role of rehabilitation professionals is to *rehabilitate* children with disabilities. According to the International Classification of Functioning, Disability and Health, the aim of rehabilitation of children with disabilities should be to maximise function, minimise incapacity and modify the environment to promote participation (WHO 2001). Rehabilitation professionals in the current study reported that rehabilitation of children with disabilities is achieved through (1) the use of *hands-on roles* where rehabilitation professionals *use different techniques to treat, facilitate, stretch* and *mobilise*; (2) *issue assistive devices* to children with disabilities; (3) *refer* children who require other services from other professionals and (4) the prescription of *home programmes* to caregivers of children with disabilities to do at home. The role of rehabilitation professionals as reported in this study, such as the hands-on role, issuing of assistive devices and initiating home programmes was also reported in other literature (Bunning *et al*. 2014; WHO 2011).

The last category of *follow-up* such as *conducting home visits* as reported in this study has also been reported in other studies. For example, one study has highlighted the importance of the home visit and home-based intervention for children with disabilities (Law *et al*. 2013).

### Limitations

There are several limitations of the study such as a small sample size of rehabilitation professionals that is not representative of the number of rehabilitation professionals available at Nkhensani Hospital. Senior physiotherapists and a speech therapist who were invited to participate in the study declined. Of all the rehabilitation professionals who participated in the study (*n* = 8), 50% (*n* = 4) belonged to the therapy assistant group and had a training of not more than 2 years. Two of the therapists were community service practitioners (interns) with less than 6 months of clinical experience. Nonetheless, the convenient sample of rehabilitation professionals who agreed to participate in the study was accepted considering the shortage of healthcare professionals all over South Africa (Gavin *et al*. 2012).

## Conclusion

From the results of the present study, it can be deduced that rehabilitation professionals play multiple roles during the rehabilitation of children with disabilities, which is similar to the role played in high-income countries (Baker *et al*. 2012; Effgen *et al*. 2007; Egilson 2011; Wiart *et al*. 2010). The various roles that rehabilitation professionals play are focused not only on the child, but also on the family. Even though it has not been mentioned as such, rehabilitation professionals in the current study used an approach that focused on both the child and the caregiver or family. This approach is similar to the family-centred approach (King, King & Rosenbaum 2004). The use of a family-centred approach together with a combination of physiotherapy and occupational therapy improve the functional status of a disabled child, as well as the satisfaction level of the caregiver (Baker *et al*. 2012). This means that health professionals should ensure that the family member(s) are active participants in the rehabilitation process in the sense that they must be given the opportunity to participate in the decision-making and goal formulation and integrate the disabled children’s management to be part of their daily routine.
